# Editorial: Challenges to EEG/MEG graph analysis and how to face them

**DOI:** 10.3389/fnins.2023.1200867

**Published:** 2023-04-24

**Authors:** Katarzyna J. Blinowska, Luiz A. Baccala, Maciej Kaminski, Koichi Sameshima

**Affiliations:** ^1^Nalecz Institute of Biocybernetics and Biomedical Engineering, Polish Academy of Sciences, Warsaw, Poland; ^2^Faculty of Physics, University of Warsaw, Warsaw, Poland; ^3^Departamento de Telecomunicações e Controle, Escola Politécnica, Universidade de São Paulo, São Paulo, Brazil; ^4^Departamento de Radiologia e Oncologia, Faculdade de Medicina, Universidade de São Paulo, São Paulo, Brazil

**Keywords:** graph analysis, connectivity, clustering, common source effect, artificial neural networks

Unveiling the interactions between brain areas and determining the topology and function of the networks connecting them is central to Neuroscience as these networks do not just integrate multiple inputs but also perform complex information processing. A large repertoire of connectivity estimators has been put forward to decipher and quantify network structure and operation; they include linear/non-linear, bivariate and multivariate methods. Determining connectivity patterns faces many technical challenges which once successfully addressed, potentially offer an appropriate descriptive brain network framework amenable to further analysis through complex network theory tools. This collection of papers portrays some different approaches to achieve that.

The paper of Strijbis et al.: “*State changes during resting state (magneto)encephalographic studies: the effect of drowsiness on spectral, connectivity, and network analyses*” used the phase lag index (PLI) and corrected amplitude envelope correlation (AECc) to generate a network topology that was appraised through the method of minimum spanning trees. Spectral analysis over 6 canonical frequency bands was also determined. Structural differences between open-eye, closed-eye and drowsiness states were examined. State and condition were far less distinct when seen through the proposed connectivity methods than when compared via spectral analysis. The best discrimination was achieved using spanning trees coupled with AECc, whereas only minimal delta band differences were noticed between drowsiness, closed-eye and open-eye states. Drowsiness had no impact on minimal spanning tree measures.

The paper of Coelho Ramos et al.: “*Spectral density-based clustering algorithms for complex networks*” investigated functional brain network changes during anesthesia. The authors applied clustering methods including k-means graph classification and model-based approaches to compare graphs of different sizes. The networks presented by the authors have very dense structure and equal connection strengths. The paper is a methodological study of different clustering approaches for binary graph comparison.

The paper by Paz-Linares et al.: “*Minimizing distortions in electrophysiological source imaging of the cortical oscillatory activity via Structured Sparse Bayesian Learning”* concerns the reconstruction of oscillating cortical sources from scalp data. The authors aimed at improving contemporary approaches by using Bayesian learning with variational approximation to the problem of setting “*a priori”* connection probabilities. The authors claim that this leads to a two order of magnitude decrease in distortion compared to other state-of-the-art methods while still being applicable to large-scale networks and not just to low-density settings as is the case of other methods.

The paper of Pidnebesna et al.: “Tackling the challenges of group network inference from intracranial EEG data” faces the problem of statistical inference and analysis of brain networks under inhomogeneous and sparse electrode placement and its differentiation across patients. The authors developed a methodological pipeline for estimating this kind of group network structure. They tested their methodology on intra-cranial data recorded for the range of visual tasks by using Directed Transfer Function (DTF)—a multivariate causal measure of connectivity– that provides directed information flow and allows representing reciprocal connections and multiple loops (Blinowska and Zygierewicz, 2022). Though tailored to the nature of the analyzed data, the methodology is easy to generalize to other network connectivity estimation methods subject to sparse inhomogeneous electrode placement constraints. In fact, despite the difficulties posed by inhomogeneous and sparse electrode placement, the paper provides new insights into the interaction between the dorsal and ventral visual streams, one of the iconic dualities in human cognition.

Within the topic's framework different approaches to network construction were examined: minimum spanning tree, clustering, application of surrogate data for construction of statistically significant group-level network from inhomogeneous data. Network architecture depended to a large degree on the chosen connectivity measure. The bivariate measures of connectivity used by Strijbis et al. and Coelho Ramos et al. due to the common drive effect portray a multitude of connections including spurious ones that blur the observed connectivity pattern (Blinowska and Kamiński, 2013; Kaminski and Blinowska, 2018). To overcome them due to the dense redundant emerging patterns, methods such as cluster analysis and spanning trees are of the essence. The latter papers can be seen as methodological contributions about different ways toward quantifying and comparing binary graphs.

The Pidnebesna et al. paper is a valuable contribution as to how to construct networks under inhomogeneous/sparse electrode placement. Its multivariate methodology takes into account connection directionality and weights leading to a viable data pipeline for estimating group network structure with robust statistical inference of brain connectivity network properties thus enabling one to monitor them while processing information.

One must stress the inherent diversity of approaches presented herein. On one hand, those relating essentially pairwise (bivariate) relationships, as in Strijbis et al. and Coelho Ramos et al. that do not account for the full features and structure of the estimated networks, and, on the other hand, those that do, as in Pidnebesna et al. and Paz-Linares et al. The latter ones, due to their multivariate nature, end up being better descriptions of the actual relationships. In fact, the use of multivariate directed representations constitute much more realistic representations.

The present collection of papers was motivated by the largely unsatisfactory present status of graph theoretical applications to neural connectivity following valid criticisms of ubiquitous “small word” descriptions (Hilgetag and Goulas, 2016; Papo et al., 2016; Hlinka et al., 2017) coupled with the almost exclusive use of binary unweighted graphs whose realism has been seriously challenged by tract-tracing experiments showing that large-scale neuronal assemblies in the brain are arranged as globally sparse hierarchical modular directed networks whose weights carry crucial biologically relevant information (Bassett and Bullmore, 2017). We hope the present set of efforts toward describing, comparing and tracking the evolution of brain networks under a variety of conditions contributes to ameliorating the present situation by examination of a range of connectivity estimation options and different types of networks employed herein. Alas their diversity still calls for an all-encompassing common formalism to tackle them. It is with these limitations in mind and acknowledging that this effort is at an early stage that we hope the present initiative may foster further developments.

To sum up the present Special Topic edition provided a promising panorama of approaches toward constructing brain networks whether they are binary or weighted directed graphs thereby opening perspectives for improving their analysis and systematic comparison.

## Author contributions

All authors listed have made a substantial, direct, and intellectual contribution to the work and approved it for publication.
